# Synthesis and molecular modeling of six novel monastrol analogues: evaluation of cytotoxicity and kinesin inhibitory activity against HeLa cell line

**DOI:** 10.1186/2008-2231-21-70

**Published:** 2013-12-19

**Authors:** Khalil Abnous, Batoul Barati, Soghra Mehri, Mohammad Reza Masboghi Farimani, Mona Alibolandi, Fatemeh Mohammadpour, Morteza Ghandadi, Farzin Hadizadeh

**Affiliations:** 1 Pharmaceutical Research Center, School of Pharmacy, Mashhad University of Medical Sciences, Mashhad, Iran; 2 School of Pharmacy, Mashhad University of Medical Sciences, Mashhad, Iran; 3 Department of Pharmacodynamics and Toxicology, School of Pharmacy, Mashhad University of Medical Sciences, Mashhad, Iran; 4 Biotechnology Research Center, School of Pharmacy, Mashhad University of Medical Sciences, P. O. Box 91775–1365, Mashhad, Iran

**Keywords:** Biginelli reaction, Dihydropyrimidine, Mitotic kinesin Eg5, Dimethylenastron

## Abstract

**Background and the purpose of the study:**

A common approach in cancer chemotherapy is development of drugs that interrupt the mitosis phase of cell division. Dimethylenastron is a known kinesin inhibitor. In this study, six novel dimethylenastron analogues (**4a-f**), in which 3-hydroxyphenyl substituent has been replaced with substituted benzylimidazolyl, were synthesized through Biginelli reaction.

**Methods:**

Six novel Biginelli compounds **(4a-f)** were synthesized through one step Biginelli reaction of imidazole aldehydes (**3a-c**), dimedone and urea or thioura. In vitro cytotoxicities of prepared compounds were investigated using MTT assay. Furthermore the ELIPA kit was implemented to study inhibitory effects of synthesized compounds on ATPase activity of kinesin by measuring of organic phosphate.

**Results:**

Our results indicated that analogue **4c** is the most toxic and analogues **4f**, **4b** and dimethylenasteron were less cytotoxic in compare with other analogues. On the other hand, analogue **4a**, **4b**, **4c** and **4e** showed stronger Kinesin inhibition as compared with analogue **4f** and dimethylenasteron. None of synthesized compounds were as potent kinesin inhibitor as Taxol. Docking analysis revealed that hydrogen bond formation and hydrophobic interactions were the key factors affecting inhibitory effects of these compounds.

**Conclusion:**

Newly synthesized compounds were found to have moderate to good cytotoxicity against HeLa cancer cell. Our results may be helpful in further design of dihydropyrimidine as potential anticancer agents.

## Background

A common approach in cancer chemotherapy is development of drugs that interrupt the mitosis phase of cell division. The mitotic spindle is an important target in cancer chemotherapy [1]. Compounds that perturb the spindle assembly checkpoint by interfering microtubule polymerization or depolymerization, arrest the cell cycle in mitosis due to prevention of the microtubule dynamics.

Nowadays, Taxol, the undisputed star, which inhibit the depolymerisation of microtubules to disassemble the mitotic spindle during cell division, is frequently used in cancer chemotherapy [2].

For the first time in 1999, Mayer *et al*., [3] identified a novel cell-permeable small molecule, named monastrol. Unlike taxol, monastrol as an antimitotic agent has not exhibited neuronal cytotoxity.

Monastrol induces a mono-astral conformation of microtubules by inhibiting the mitotic kinesin Eg5 [4,5].

**Figure 1:**
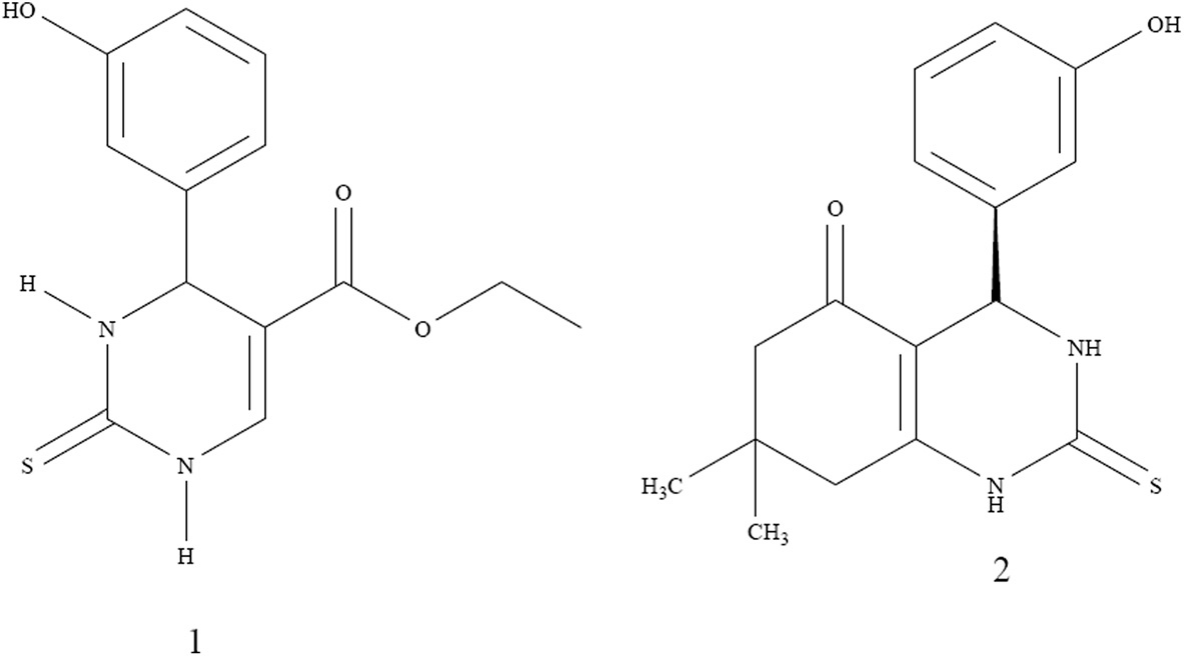
Monastrol (1) and dimethylenastron (2) structures.

**Figure 2:**
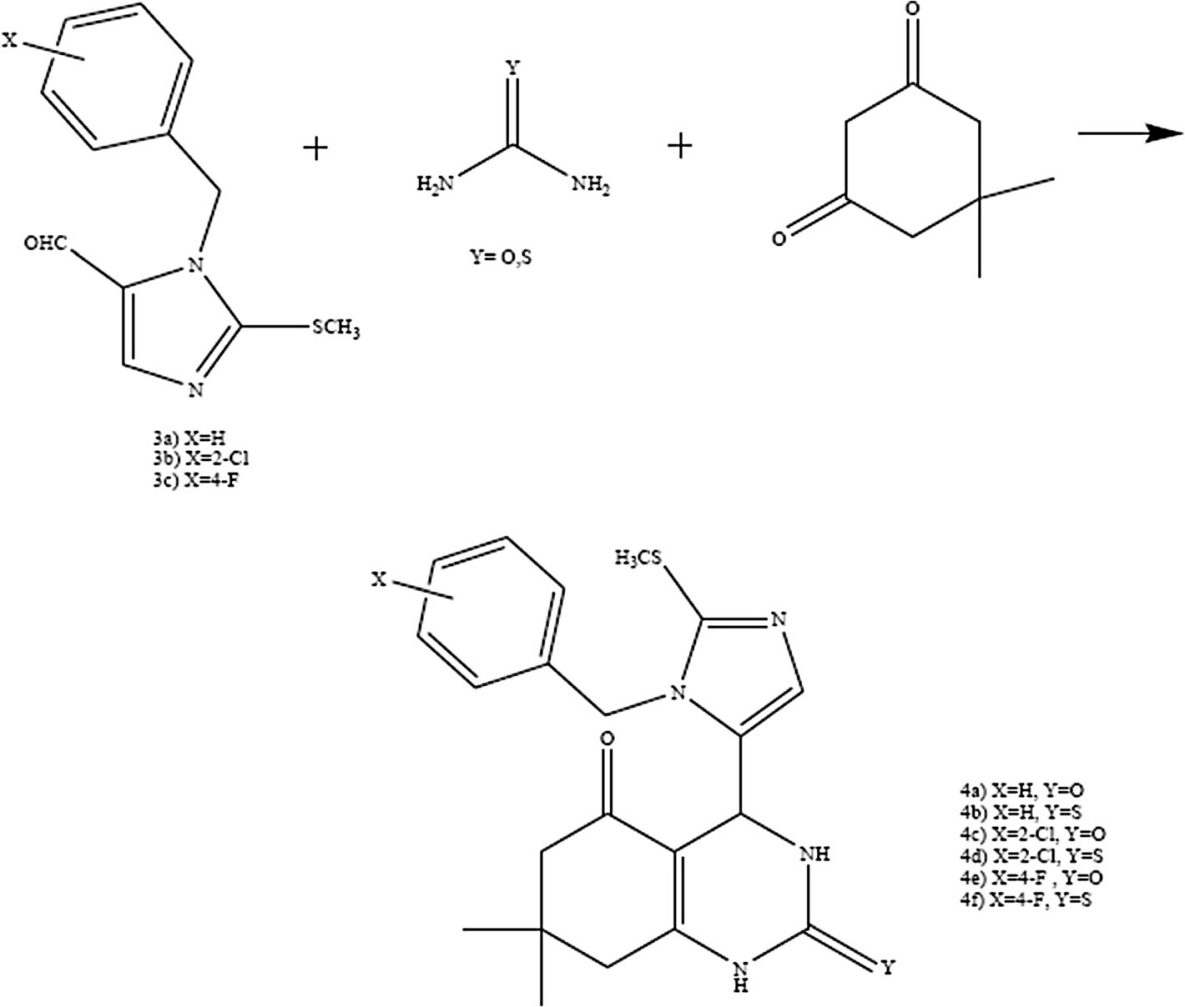
Synthesis of 4-imidazolyl tetrahydroquinazolines (4a-f) under Biginelli condition.

Exploration of monastrol, commenced a new stage in Biginelli 3,4-dihydropyrimidine-2(1H)-one chemistry. Although many researches have been devoted to reveal the anti-mitotic mechanism of monastrol in the cell cycle [6-8], there are few examples concerning the anticancer activity [9-11]. Leizerman *et al.*[12] described the antiproliferative effect of monastrol on AGS and HT-29 cell lines as compared with taxol. Since the antimitotic activity of monastrol is not very high, structural variants could be verified to have better activity. Russowsky *et al.*[13], investigated the differential antiproliferative activity of monastrol, oxo-monastrol and oxygenated analogs on seven human cancer cell lines. In another study, more potent analogs of monastrol such as dimethylenastron [14] and quinazoline-2(1H)-thione [15] (Figure 1) were provided by skeleton modifications of monastrol in the parent ring by annelation across the C-5–C-6 bond. Notable work has also been dedicated to delineate the structure-activity relationship in the monastrol derivatives [16]. In this work six novel compounds (**4a-f**) were synthesized through Biginelli reaction in which hydroxyphenyl at C-4 position in dimethylenastron has been replaced with substituted benzylimidazolyl (Figure 2).

## Methods

### Chemistry

Melting points were determined using an Electrothermal Capillary apparatus and are uncorrected. ^1^H-NMR spectra were recorded using Bruker AC-80 NMR spectrometer. The chemical shift values are on δ scale and the coupling constant values (J) are in ppm relative to tetramethylsilane as internal standard. Errors of elemental analyses were within ±0.4% of theoretical values.

The desired compounds were synthesized by the reactions outlined in Figure 2. Imidazole aldehydes [**3a-c**] was synthesized as described previously [17].

### General procedure for synthesis of 4a-f

The suspension of **3a-c** (2 mmoles)**,** dimedone (2 mmoles) and urea or thiourea (2.4 mmoles) in TMSCl (0.25 ml), DMF (0.8 ml) and acetonitrile (1.6 ml) was stirred for 4 h. The solid was separated by centrifugation and washed with distilled water followed by methanol. The residue was completely dried to give compound **4**.

### *4-[1-benzyl-2-(methylthio)-1H-imidazol-5-yl)- 3,4,7,8- tetrahydro- 7,7 dimethyl- quinazoline-2,5-(1H,6H)-diones (4a)*

This compound was obtained in 44% yield; mp 169°C; ^1^H-NMR (DMSO-d_6_): 9.5(s, 1H, NH), 8 (s, 1H, NH), 7.82–6.94 (m, 6H, arom, H-imidazole), 6.21–5.44 (3H, CH_2_N, C-H quinazoline), 2.91 -2.52 (m, 7H, SCH_3_, 6,8,CH_2_ quinazoline), 1.111-0.93 (m, 6H,CH_3_ quinazoline). Anal. Calcd for C_21_H_24_N_4_O_2_S: C, 63.61; H, 6.10; N, 14.13. Found: C, 63.48; H, 6.07; N, 14.07.

### *4-[1-benzyl-2-(methylthio)-1H-imidazol-5-yl)-1,2,3,4,7,8- hexahydro- 7,7 dimethyl-2-thio oxoquinazoline-5-(6H)-one (4b)*

This compound was obtained in 36% yield; mp 157°C; ^1^H-NMR (DMSO-d_6_): 9.5 (s, 1H, NH), 8.00 (S, 1H, NH), 7.62 –6.95 (m, 6H, arom, H-imidazole), 5.54 (3H, CH_2_N, C-H quinazoline), 2.93 - 2.54 (m, 7H, SCH_3_, 6,8,CH_2_ quinazoline) 1.11-0.83 (m, 6H, CH_3_ quinazoline). Anal. Calcd for C_21_H_24_N_4_OS_2_: C, 63.13; H, 5.86; N, 13.58. Found: C, 61.27; H, 5.88; N, 13.63.

### *4-[1-(2-chlorobenzyl)-2-(methylthio)-1H-imidazol-5-yl]-3,4,7,8- tetrahydro- 7,7 dimethyl quinazoline-2,5-(1H,6H)-diones (4c)*

This compound was obtained in 61% yield; mp 149°C; ^1^H-NMR (DMSO-d_6_): 9.5(s, 1H, NH), 8.1(s, 1H, NH), 7.43-6.02 (m, 5H, arom, H-imdazole), 5.53 (3H, CH_2_N, C-H quinazoline), 2.82 -2.53 (m, 7H, SCH_3_, 6,8- CH_2_ quinazoline), 1.11-0.74 (m, 6H, CH_3_ quinazoline). Anal. Calcd for C_21_H_23_ClN_4_O_2_S: C, 58.53; H, 5.38; N, 13.00. Found: C, 58.68; H, 5.40; N, 12.94.

### *4-[1-(2-chlorobenyl)-2-methylthio-1H-imidazol-5-yl]-1,2,3,4,7,8- hexahydro-7,7 dimethyl-2-thio oxoquinazoline-5-(6H)-one (4d)*

This compound was obtained in 58.1% yield; mp 142°C; ^1^H-NMR (DMSO-d_6_): 9.5 (s, 1H,NH), 8.13 (s, 1H, NH) 7.63-6.41 (m, 5H, arom, H-imdazole) 5.52 (3H, CH_2_N, C-H quinazoline), 2.83 -2.45 (m, 7H, SCH_3_, 6,8- CH_2_ quinazoline), 1.23-1.02 (m, 6H, CH_3_ quinazoline). Anal. Calcd for C_21_H_23_ClN_4_OS_2_: C, 56.42; H, 5.19; N, 12.53. Found: C, 56.31; H, 5.21; N, 12.45.

### *4-[1-(4-fluorobenzyl)-2-(methylthio)-1H-imidazol-5-yl]-3,4,7,8 – tetrahydro-7,7 dimethyl quinazoline-2,5-(1H,6H)-diones (4e)*

This compound was obtained in 68.7% yield; mp 82°C; ^1^H-NMR (DMSO-d_6_): 9.52 (s, 1H, NH), 7.91 (s, 1H, NH), 7.14- 7.11 (m, 5H, arom, H-imdazole), 5.41-5.23 (3H, CH_2_N, C-H quinazoline), 2.81- 2.05 (m, 7H, SCH_3_, 6,8- CH_2_ quinazoline), 1.22 -0.92 (m, 6H, CH_3_ quinazoline). Anal. Calcd for C_21_H_23_FN_4_O_2_S: C, 60.85; H, 5.59; N, 13.52. Found: C, 60.80; H, 5.61; N, 13.46.

### *4-[1-(4-fluorobenzyl)-2-methylthio-1H-imidazol-5-yl]-1,2,3,4,7,8 -hexahydro-7,7-dimethyl-2-thiooxoquinazoline-5-(6H)-one (4f)*

This compound was obtained in 58% yield; mp 149°C; ^1^H-NMR (DMSO-d_6_): 9.5 (s, 1H, NH), 8.1 (s, 1H, NH), 7.33-7.10 (m, 5H, arom, H-imdazole), 5.43 (3H, CH_2_N, C-H quinazoline), 2.85- 2.12 (m, 7H, SCH_3_, 6,8-CH_2_ quinazoline), 2.81 -2.43 (m, 3H, SCH_3_), 1.22-0.94 (m, 6H, CH_3_ quinazoline). Anal. Calcd for C_21_H_23_FN_4_OS_2_: C, 58.58; H, 5.38; N, 13.01. Found: C, 58.61; H, 5.39; N, 13.06.

### Docking

The X-ray crystal structure of Eg5-enastron complex (Protein Data Bank ID: 2X7C) was obtained from the Protein Data Bank. The three-dimensional structures of the derivatives were constructed using molecular mechanic force field (MM+), pre-optimization and AM1 semiemperical calculation in Hyperchem 7 software. The final corrected PDB file of the protein and synthesized analogs were submitted to AutoDock tools in order to run docking process. Docking studies were performed by AutoDock software Version 4. Searching was conducted within a specified 3D docking box (40 angstrom in all aspects) around enastron and the number of GA runs adjusted to 20 using Lamarckian genetic algorithm and all other parameters set as default. At the final stage through the docked structures of all analogs, best conformation was selected and saved as PDB file.

PDB files of best docked analogs along with Eg5 protein were submitted to MOE 2007.11 (License purchased from Chemical Computing Group by Mashhad University Medical Sciences, http://www.chemcomp.com/ for preparing figures and running protein ligand interaction fingerprint (PLIF).

The virtual physicochemical parameters of the synthesized compounds were also determined using MOE 2007.11 software.

### Cell culture

HeLa cell line was obtained from National Cell Bank of Iran, Pasteur Institute of Iran. Cells were cultured in DMEM medium (Gibco, USA) supplemented with 10% (v/v) heat-inactivated fetal bovine serum (Gibco, USA), 100 U/ml penicillin (Biosera, UK), and 100 mg/ml streptomycin (Biosera, UK) at 37°C in a humidified atmosphere (95%) containing 5% CO_2._

### Cell viability

Cytotoxic effects of synthesized compounds were determined using the MTT [3-(4,5-dimethylthiazol-2-yl)-2,5-diphenyltetrazolium bromide] assay [18-20]. Briefly, 5000 HeLa cells/well were seeded in a 96-well plate and cultured overnight. Different concentrations of synthesized compounds were added to each well. After incubation for 24 h, cells were treated with MTT solution (final concentration 0.5 mg/ml; Sigma, USA) for 4 h at 37°C. Then, the medium was removed, and the purple formazan crystals were dissolved in 150 μl dimethylsulfoxide (Merck, Germany). Absorbance was measured at 545 nm (630 nm as a reference) in Synergy H4 Hybrid Multi-Mode Microplate Reader (Biotek, Model: H4MLFPTAD). IC_50_ was calculated using CalcuSyn (BioSoft).

### Kinesins activity assay

Briefly MT ELIPA master mix was prepared using 2 ml of reaction buffer containing 15 mM PIPES pH 7, 5 mM MgCl_2_, 160 μl of 1 μg/μl tubuline solution in reaction buffer, 480 μl of ELIPA reagent 1 containing 1 mM 2-amino-6-mercapto-7-methylpurine riboside (MESG) and 24 μl of ELIPA reagent 2 containing 0.1 units/μl of purine nucleoside phosphorylase (PNP). Final reaction mixture was prepared by adding 75 μl of ELIPA master mix, 1 μl of 2.5 mg/ml kinesin heavy chain Motor (Cytoskeleton, Cat # KR01-A) and 20 μl of different concentrations of synthesized inhibitors and Taxol. Reaction was started by adding 8 μl of 100 mM ATP stock solution (Sigma, Cat # A3377). Absorbance of each well was recorded on a kinetic mode at λ_360_ nm wavelength using Synergy H4 Hybrid Multi-Mode Microplate Reader (Biotek, Model: H4MLFPTAD). Phosphate standard curve was constructed by adding 0–25 μl of 0.5 mM phosphate stock, to ELIPA master mix (Without tubulin). Activity of kinesin was reported as nmole Pi/min/2.5 μg kinesin.

### Statistical analysis

Data are expressed as mean ± SD. Statistical analyses were performed with ANOVA followed by Tukey–Kramer test to compare the differences between means. Differences were considered statistically significant when P < 0.05.

## Results and discussion

### Chemistry

In this study the new dimethylenastron derivatives (**4a-f**) were produced by substitution of 3-hydroxyphenyl in dimethylenastron with substituted benzyl imidazolyl under Biginelli condition.

The purity of the compounds was checked by TLC and melting points. The structure of the compounds was confirmed on the basis of its ^1^H NMR spectral data and elemental analyses. All spectral data are in accordance with assigned structures. In IR spectra, N-H and C-O stretching bands were observed at spectra expected values. In the ^1^H NMR spectra, methyl protons were seen at 0.90-1.00 ppm as separated singlets. Aromatic, methylene, methine and NH protons were found at expected values.

### Docking analysis

**Figure 3:**
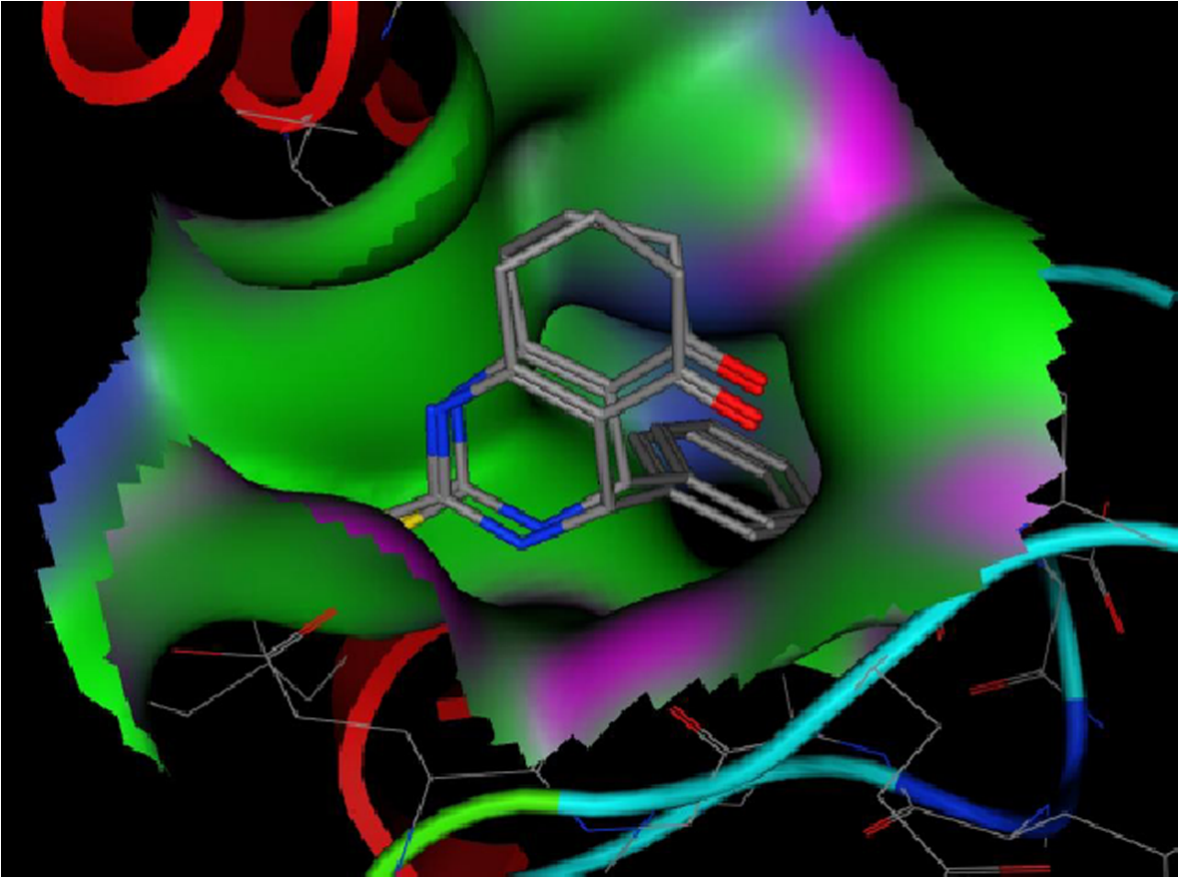
Docked and co-crystalized enasteron in Eg5 enzyme.

Accuracy of docking protocol was examined by docking enastron in active site of Eg5 enzyme. Figure 3 shows docked enastron and co-crystallized one in almost same position among the receptor (RMSD = 1.24Ǻ) that confirmed validation of docking protocol. All dimethylenastron derivatives were docked into active site of Eg5.

**Figure 4:**
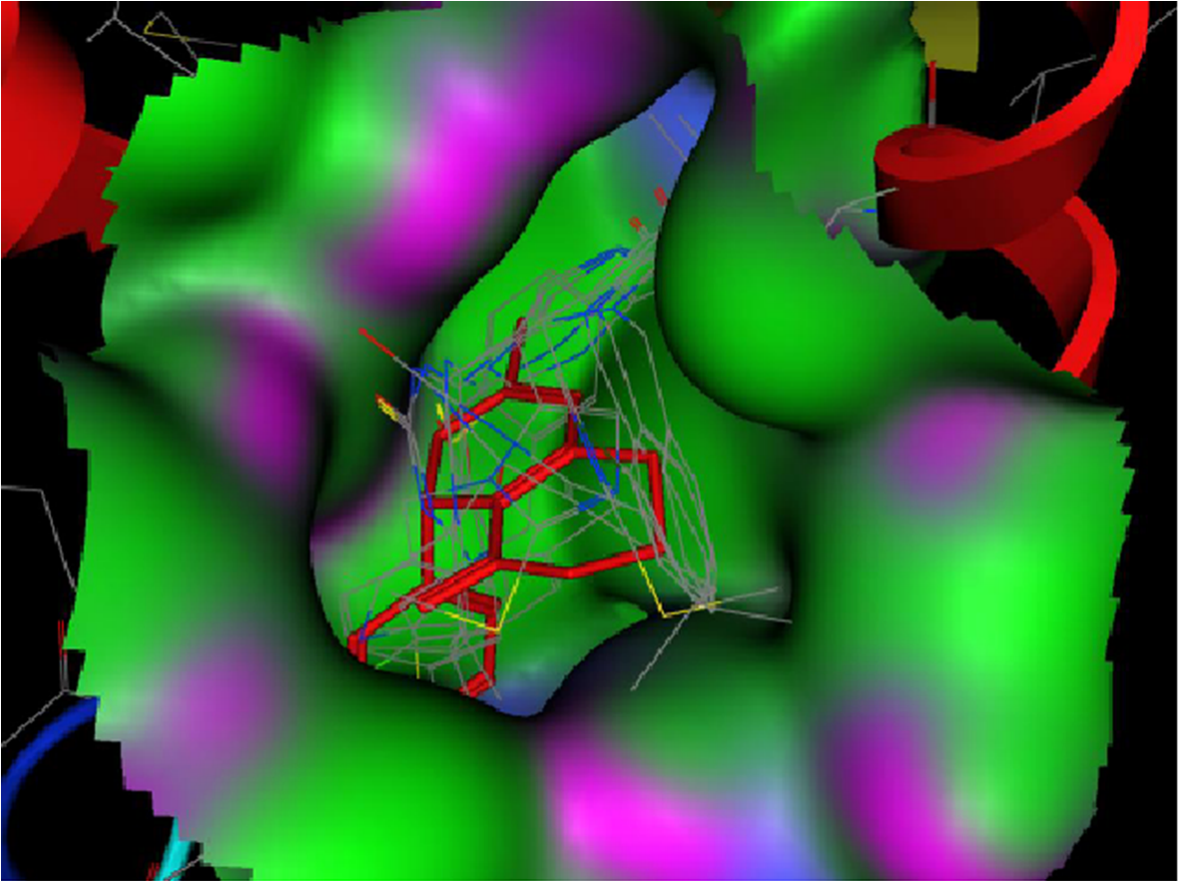
**Map surface of docked analogs in active site of enzyme (Green: hydrophobic; Violet: H bonding; Blue: mild polar).** Crystallized enasteron is indicated by red and stick lines.

Table 1 shows estimated free energy of binding and calculated ki of synthesized compounds extracted from docking studies, these data in addition to Figure 4 which indicates synthetic ligands were in suitable position between active site of enzyme approve suitable interaction between ligands and protein.

**Table 1:** Cytotoxicity on HeLa cell line (n = 5), inhibition of Kinesin activity (n = 2) and docking results of synthesized analogues 4a-f

**Compound**	X*	Y*	**IC**_**50 **_**(μg/ml) ± SD on HeLa cell line**	**IC**_**50 **_**(μg/ml) for Kinesin inhibition**	Estimated** free energy of binding	Calculated*** Ki (nM) for Kinesin inhibition
**4a**	H	O	210 ± 21	72	-8.94	281.5 nM
**4b**	H	S	301 ± 22	77	-8.67	438.63 nM
**4c**	2-Cl	O	98 ± 19	79	-9.39	130.76 nM
**4d**	2-Cl	S	ND**	ND**	-8.83	334.27 nM
**4e**	4-F	O	110 ± 28	96	-8.55	536.29 nM
**4f**	4-F	S	339 ± 23	198	-8.52	573.33 nM
dimethylenastrone	-	-	338 ± 26	210	-8.72	409.07 nM
Taxol	-	-	ND**	7	ND	ND

*X and Y were represented in Figure 2.

**Not determined.

***Docking results.

**Figure 5:**
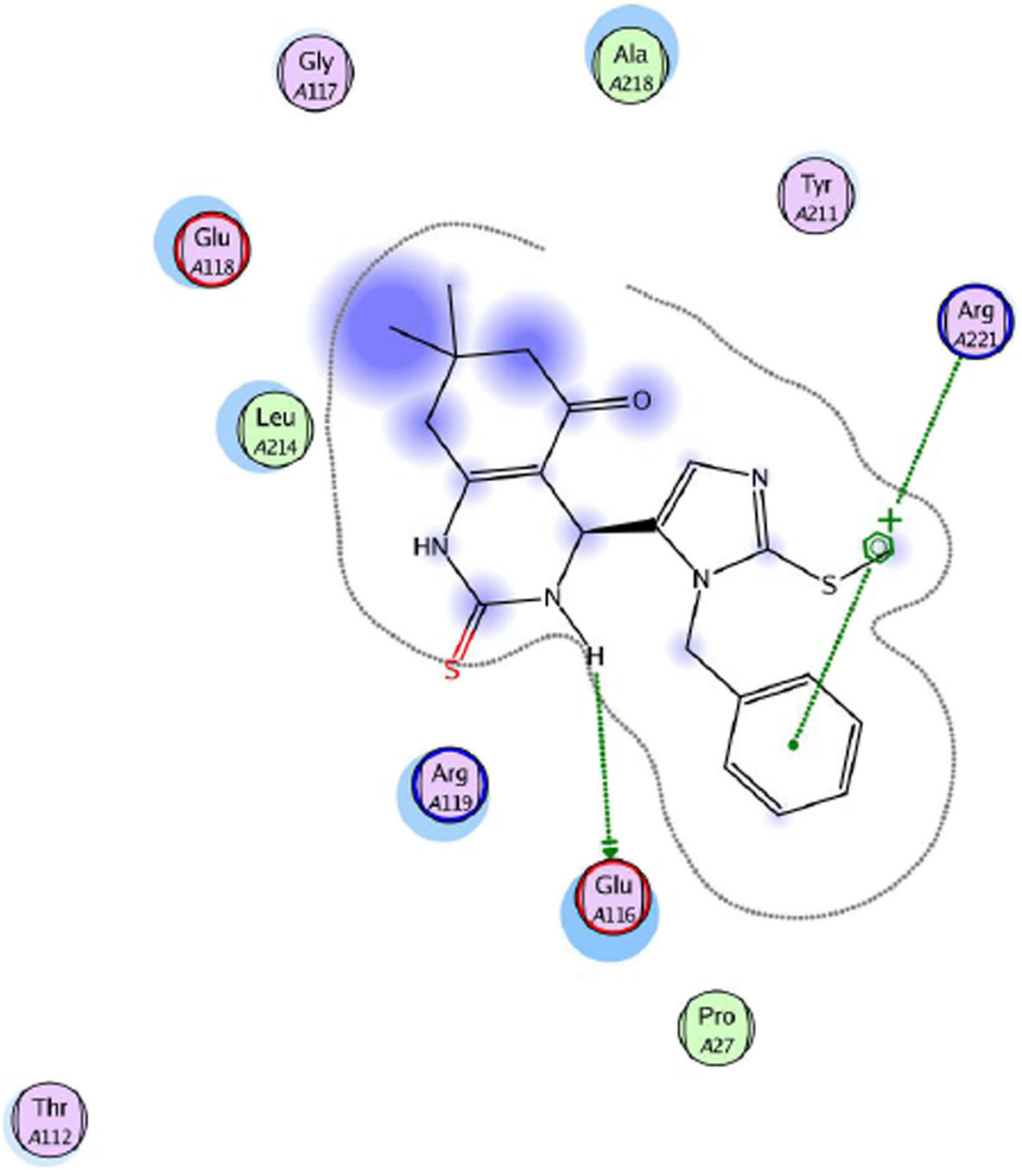
**2D graph of interactions between synthesized compound 4b and protein made by LigX module of MOE software.** In the 2D graphs hydrophobic/aromatic residues are colored in green, whereas polar amino acids are shown in magenta. H-bonds and all π-stacking interactions are represented as green dotted lines. The active site contour is also shown.

According to PLIF, the most consistence interaction is H-bound between side chain of Glu116 and nitrogen atom on quinazoline ring. Compounds **4a**, **4b**, **4c** and **4d** have shown mentioned interaction and a backbone H-bound donor interaction was also detected between Glu118 and nitrogen atom in imidazolic ring of compound **4a**. On the other hand by calculating ligand-protein interactions using LigX module in MOE software, except these H-bound interactions, surface contact interactions (arene-cation) between imidazolic ring of ligands and Arg 221 were identified. (Figure 5) Figure 5 represents 2D graph of interactions between synthesized compound **4b** and Eg5 protein calculated by LigX module.

Docking analysis revealed that the all compounds interacted with Eg5 in good manner and confirms the importance of H-bound donor group in quinazoline ring and the role of imidazole ring as well as benzene ring in surface contact interaction on antiproliferative effects of synthesized compounds.

### Effects of synthesized compounds on cell viability

Cytotoxicity of synthesized compounds was evaluated using MTT assay (Table 1). HeLa cells were incubated with different concentrations of newly synthesized compounds for 24 h and cytotoxicities were evaluated using MTT assay. Our data showed that analogue **4c** (X = 2-Cl Y = O, IC_50_: 98 ± 19 μg/ml) was the most toxic compound among the newly synthesized compounds. Analogue **4f** (X = 4-F Y = S, IC_50_: 339 ± 23 μg/ml), **4b** (X = H Y = S, IC_50_: 301 ± 22 μg/ml) and dimethylenasteron (IC_50_: 338 ± 26 μg/ml) showed less cytotoxicities.

The significant difference between cytotoxicity of analogues **4f** (X = 4-F Y = S, IC_50_: 339 ± 23 μg/ml) and **4e** (X = 4-F Y = O, IC_50_: 110 ± 28 μg/ml) or analogue **4b** (X = H Y = S, IC_50_: 301 ± 22 μg/ml) and **4a** (X = H Y = O, IC_50_: 210 ± 21 μg/ml)demonstrated the essential role of carbonyl group at position C2 of tetrahydro-quinazoline on antiproliferative effect of synthesized compounds.

Meanwhile there was no big difference between cytotoxicity of compound **4c** (X = 2-Cl Y = O, IC_50_: 98 ± 19 μg/ml) and **4e** (X = 4-F Y = O, IC_50_: 110 ± 28 μg/ml); they had so much better cytotoxic effect in comparison with compounds **4b** (X = H Y = S, IC_50_: 301 ± 22 μg/ml) and **4a** (X = H Y = O, IC_50_: 210 ± 21 μg/ml). The obtained results illustrated the presence of an electron withdrawing groups (F or Cl) on the benzylimidazolyl substituent at position C4 of tetrahydro-quinazoline structure could increase the cytotoxicity of prepared compounds.

### Effects of synthesized compounds on Kinesin activity

Kinesins operate by utilizing the energy of ATP hydrolysis, generating Pi, to move along microtubule (MT) substrates. To determine activity of kinesins, rate of Pi production was measured using ELIPA (Enzyme Linked Inorganic Phosphate Assay) Biochem kit (Cytoskeleton, cat # BK060). The assay was based on an absorbance shift (330–360 nm) that occurred when 2-amino-6-mercapto-7-methylpurine ribonucleoside (MESG) was catalytically converted to 2-amino-6-mercapto-7-methylpurine in the presence of inorganic phosphate (Pi). The reaction was catalyzed by purine nucleoside phosphorylase (PNP). One molecule of Pi produces one molecule of 2-amino-6-mercapto-7-methylpurine in an essentially irreversible reaction. Thus, the absorbance at 360 nm is directly proportional to the amount of Pi. Our data (Table 1) showed that analogue **4a** (X = H Y = O, IC_50_: 72 μg/ml), **4b** (X = H Y = S, IC_50_: 77 μg/ml**)**, **4c** (X = 2-Cl Y = O, IC_50_: 79 μg/ml) and **4e** (X = 4-F Y = O, IC_50_: 96 μg/ml) were stronger Kinesin inhibitor as compared with analogue **4f** (X = 4-F Y = S, IC_50_: 198 μg/ml) and dimethylenasteron (IC_50_: 210 μg/ml. None of our compounds were able to inhibit kinesin as much as Taxol (IC_50_: 7 μg/ml).

According to kinesin inhibitory effect of analogues **4f** and **4e**, the carbonyl group at C2 position of tetrahydro-quinozoline was crucial in kinesin inhibitory activities of synthesized compounds. On the other hand, our results suggested that the presence of an electron withdrawing group in benzene ring of benzylimidazolyl at position C4 of tetrahydro-quinazoline structure had no significant effect either positive or negative on kinesin inhibitory activity of synthesized compounds.

## Conclusion

In this study a series of six novel dimethylenastron analogs based on Biginelli reaction were synthesized with IC_50_ in the range of 98 to 210 μg/mL against HeLa cell line. Compared with dimethylenastron, these new series were found to have stronger antiproliferative activity against HeLa cell line. Structural-activity relationship study highlighted the important role of carbonyl substitution at C2 position of tetrahydro-quinazoline structure in antiproliferative and kinesin inhibitory effect of newly synthesized compounds. The reported results could be helpful in developing potential formulation of new anticancer drugs.

## Abbreviations

DMF: Dimethylformamide; ELIPA: Enzyme linked inorganic phosphate assay; IR: Infrared; MESG: 2-amino-6-mercapto-7-methylpurine ribonucleoside; MTT: 3-(4,5-Dimethylthiazol-2-yl)-2,5-diphenyltetrazolium bromide; MT: Microtubule; NMR: Nuclear magnetic resonance; PLIF: Protein ligand interaction fingerprint; PNP: Purine nucleoside phosphorylase; RMSD: Root-mean-square deviation; TLC: Thin layer chromatography; TMSCL: Trimethylsilyl chloride.

## Competing interests

The authors declare that they have no competing interests.

## Authors’ contributions

KA: Supervision of biological studies including cytotoxicity (MTT) and Kinesin inhibition assay. BB: performed synthesis of the target compounds. SM: Collaboration in biological studies, MRMF: Performed the cytotoxic tests and Kinesin Assay. MA: manuscript draft preparation and Collaboration in biological studies. FM: performed synthesis and identifying of the target compounds. MG: performed molecular modeling studies. FH: supervision of design and synthesis of target compounds and manuscript preparation. All authors read and approved the final manuscript.
